# Clinical manifestations and ultrasonographic features of lobular endocervical glandular hyperplasia: a retrospective study of 135 patients

**DOI:** 10.1080/07853890.2026.2673627

**Published:** 2026-06-01

**Authors:** Xiuying Chen, Zhiyun Xue, Sulan Zhao, Li Sun, Bin Li

**Affiliations:** Obstetrics and Gynecology Hospital of Fudan University, Shanghai, China

**Keywords:** Diagnosis, cervical cystic lesion, LEGH, ultrasonography

## Abstract

**Background:**

Lobular endocervical glandular hyperplasia (LEGH) is a precursor to malignant lesions of the endocervix. However, precise histopathological sampling for early detection remains a challenge. This study aimed to review the clinical features of LEGH and particularly focus on its ultrasonographic characteristics.

**Patients:**

A retrospective analysis was conducted on 135 patients with LEGH (including those with or without concurrent malignancy). Clinical data, preoperative diagnostic evaluations, and histopathological findings were systematically collected and analyzed. Concurrently, we focused on the ultrasonographic features of LEGH.

**Results:**

Symptoms of watery vaginal discharge were clinically documented in 63.0% (85/135) of the patients in this study cohort. Cytological analysis revealed negative results in 88.1% (119/135) of the specimens. Sonographic evaluation identified multicystic cervical lesions or masses and occupation of the uterine cavity in 59.3% (80/135) of cases, whereas magnetic resonance imaging (MRI) demonstrated superior detection capability in 92.9% (104/112) of the examined patients. Sonographic examination showed heterogeneous echogenic areas of various sizes in the upper part of the cervix. The cystic lesions presented with enhanced internal and peripheral echogenicity, and the boundary was not clear. Ultrasonographic images also revealed absent or sparse blood flow signals in LEGH. The detection rate of vaginoscopic biopsy was of 48.5% (33/68).

**Conclusion:**

The preoperative diagnostic workup for LEGH requires the integration of clinical manifestations, cervical cytology, sonographic evaluation, and pelvic MRI. Sonographic evaluation demonstrated heterogeneous echogenicity, echogenic enhancement, obscure boundaries, and no significant blood flow signals as the defining features of LEGH.

## KEY MESSAGES

LEGH is an HPV-independent cervical lesion that is easily missed by conventional cytology and HPV testing; however, it can be identified through characteristic ultra­sonographic features such as heterogeneous echogenicity with multiple cysts, peripheral echogenic enhancement, obscure boundaries, and sparse internal vascularity.

Preoperative diagnosis requires a high index of suspicion based on symptoms such as watery discharge, and should be confirmed using targeted biopsy methods such as hysteroscopy and/or conization, as conventional vaginoscopic biopsy has a high miss rate for these often high-located lesions.

## Introduction

Lobular endocervical glandular hyperplasia (LEGH) is a hyperplastic lesion of the endocervical glands that was initially reported as a benign disease histologically similar to minimal deviation adenocarcinoma (MDA) in 1999 [1]. A subset of LEGH is considered a precursor to Gastric-type mucinous carcinoma and MDA [2,3]. Patients with LEGH may complain of vaginal discharge; however, as they often test negative for human papillomavirus (HPV) and cervical cytology screening, the condition is easily overlooked in clinical practice, leading to missed diagnoses. The ‘cosmos pattern’ as characteristic imaging in magnetic resonance imaging (MRI) has been reported for LEGH, and ‘microcystic pattern’ also suggesting LEGH or other malignant lesions [4,5]. However, LEGH lesions are easily missed on ultrasound examinations [6], and most physicians do not recognize the ultrasonographic characteristics of LEGH. A definitive diagnosis of LEGH or malignant lesions is based on histopathological assessments. In the present study, we retrospectively investigated the clinical data of LEGH cases with or without concurrent malignancy and focused on its ultrasonographic characteristics to raise awareness of LEGH and improve the comprehensive management of LEGH.

## Patients

The study included 135 cases of LEGH with or without concurrent malignancy (average age: 43.44 ± 10.51 years) that were histologically confirmed at the Obstetrics and Gynecology Hospital of Fudan University between January 2020 and December 2023. Patient information, such as age, gravida, parity, menopausal state, symptoms, preoperative assessment, diagnostic method, and pathology after hysterectomy, was collected. All the patients underwent Pap smear tests, HPV testing, and transvaginal sonography at the initial visit. When LEGH or malignancy was suspected, the patient underwent MRI examination. Whether vaginoscopic biopsy, cervical conization, hysteroscopic biopsy, or cervical conization combined with hysteroscopic biopsy was performed depended on the clinician’s judgment. When LEGH or malignancy was confirmed by pathology, hysterectomy was recommended unless the patient still had fertility needs and refused hysterectomy. This study was approved by the Human Ethics Review Committee of the Obstetrics and Gynecology Hospital of the Fudan University (2025-157). Informed consent was obtained from all participants prior to data collection.

## Results

Table 1 presents the patients’ background, including age, gravidity, parity, menopausal status, and symptoms. Of the 135 patients, 17.0% (23/135) were postmenopausal. The majority of patients (43.7%, 59/135) were between 40 and 49 years of age. The total prevalence of watery discharge was 63.0% (85/135). Of the 135 patients, 25.2% (34/135) had Peutz–Jeghers syndrome (PJS). Carcinoma *in situ* or malignancy was present in 20.0% (27/135) of patients.

**Table 1.** Patients’ background of cases in the group.

Characteristics	Number
**Age**	
**(years)**	
<30	13
30–39	31
40–49	59
50–59	26
≥60	6
**Gravida**	
0–1	40
2–3	72
≥3	23
**Para**	
0	33
1	75
≥2	27
**Menopause**	
Pre	112
Post	23
**Symptom**	
Watery discharge	85
Vaginal bleeding	1
None	49
**PJS**	
Yes	34
No	101
**Pathological diagnosis**	
Only LEGH	57
Atypical LEGH	51
AIS combined with LEGH	14
Gastric-type mucinous carcinoma or MDA combined with LEGH	13

**Figure 1.**
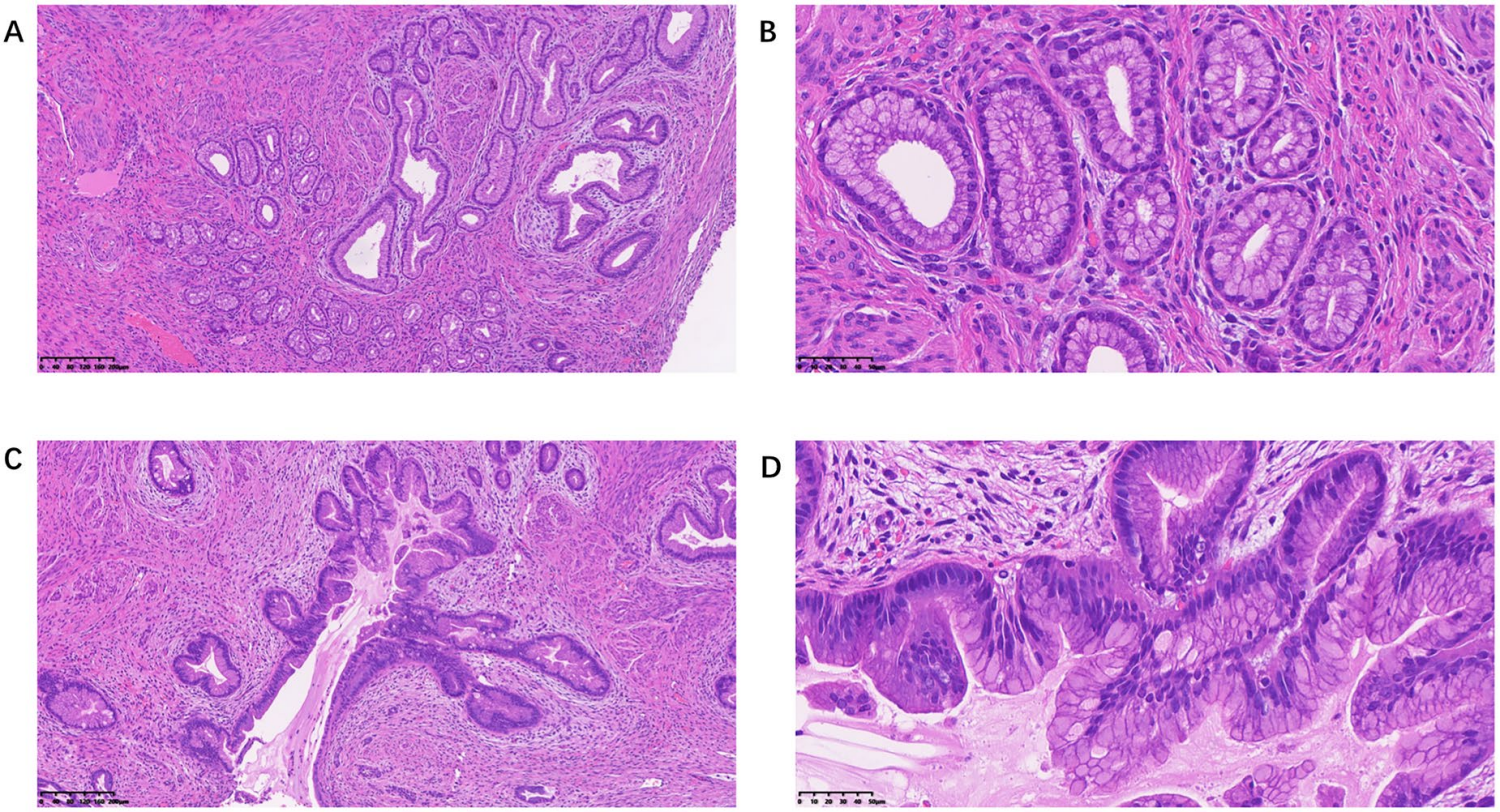
Photomicrographs of LEGH (A and B), LEGH with atypia (C and D). Histologically, LEGH is characterized by lobular distribution of small glands lined by normal-looking endocervical cells without nuclear atypia, often locating in the upper portion of the endocervical canal (A, B). LEGH with cytological and architectural atypia without stromal invasion, and it was termed atypical LEGH (C and D).

Table 2 presents the patients’ preoperative assessments, including cervical cytology, HPV, ultrasound, MRI, different diagnostic methods, and pathology after hysterectomy. A total of 119 (88.1%) patients showed NILM upon cytological examination. A total of 118 (87.4%) patients tested negative for HPV infection. Although 17 patients were positive in the HPV tests, they all had cervical intraepithelial neoplasia. Ultrasonography revealed multiple cervical cysts or masses and occupation in the uterine cavity in 59.26% of cases (80/135), while lesions were found in 92.9% (104/112) of cases on MRI. Magnetic resonance imaging (MRI) revealed multiple cervical cysts, abnormal signal, suspected LEGH, or suspected malignancy; however, there were still eight patients without any anomaly discovery. On histology (Figure 1), LEGH exhibits a lobular architecture composed of small glands lined by endocervical cells with a normal morphology and no nuclear atypia; it is typically situated in the upper endocervical canal. The term ‘atypical LEGH’ applies to lesions that show cytological or architectural atypia but no evidence of stromal invasion. Confirmed or suspected LEGH or greater lesions were considered positive diagnoses, and 35 cases missed diagnosis in 68 patients who underwent vaginoscopic biopsy. There were 31 cases diagnosed by cervical conization, 37 by hysteroscopic biopsy, and 40 by cervical conization combined with hysteroscopic biopsy. Hysterectomy was not performed in 35 patients. According to the pathology after hysterectomy in 100 patients, 12 patients underwent pathological upgrading, and 25 cases were defined through serendipitous discoveries after hysterectomy.

**Table 2.** Patients’ preoperative evaluation and pathological diagnosis of cases in the group.

Characteristics	Number
**Cervical cytology**	
NILM	119
ASC	7
AGC	7
LSIL/HISL	2
**HPV**	
Negative	118
Positive	17
**Ultrasound**	
Cervical multiple cyst/mass	71
Occupation in uterine cavity	9
Normal	55
**MRI**	
Cervical multiple cyst	26
Abnormal signal	16
Suspected LEGH	40
Suspected malignancy	22
Normal	8
Never checked	23
**Vaginoscopic biopsy**	
Confirmed or suspected LEGH or greater lesion	33
No lesion	35
Never checked	67
**Diagnostic method**	
Cervical conization	31
Hysteroscopic biopsy	37
Cervical conization combined with hysteroscopic biopsy	40
**Pathology after hysterectomy**	
Pathological consistent	63
Pathological upgrading	12
Serendipitous discoveries after hysterectomy	25
Hysterectomy rejected by the patient	35

**Figure 2.**
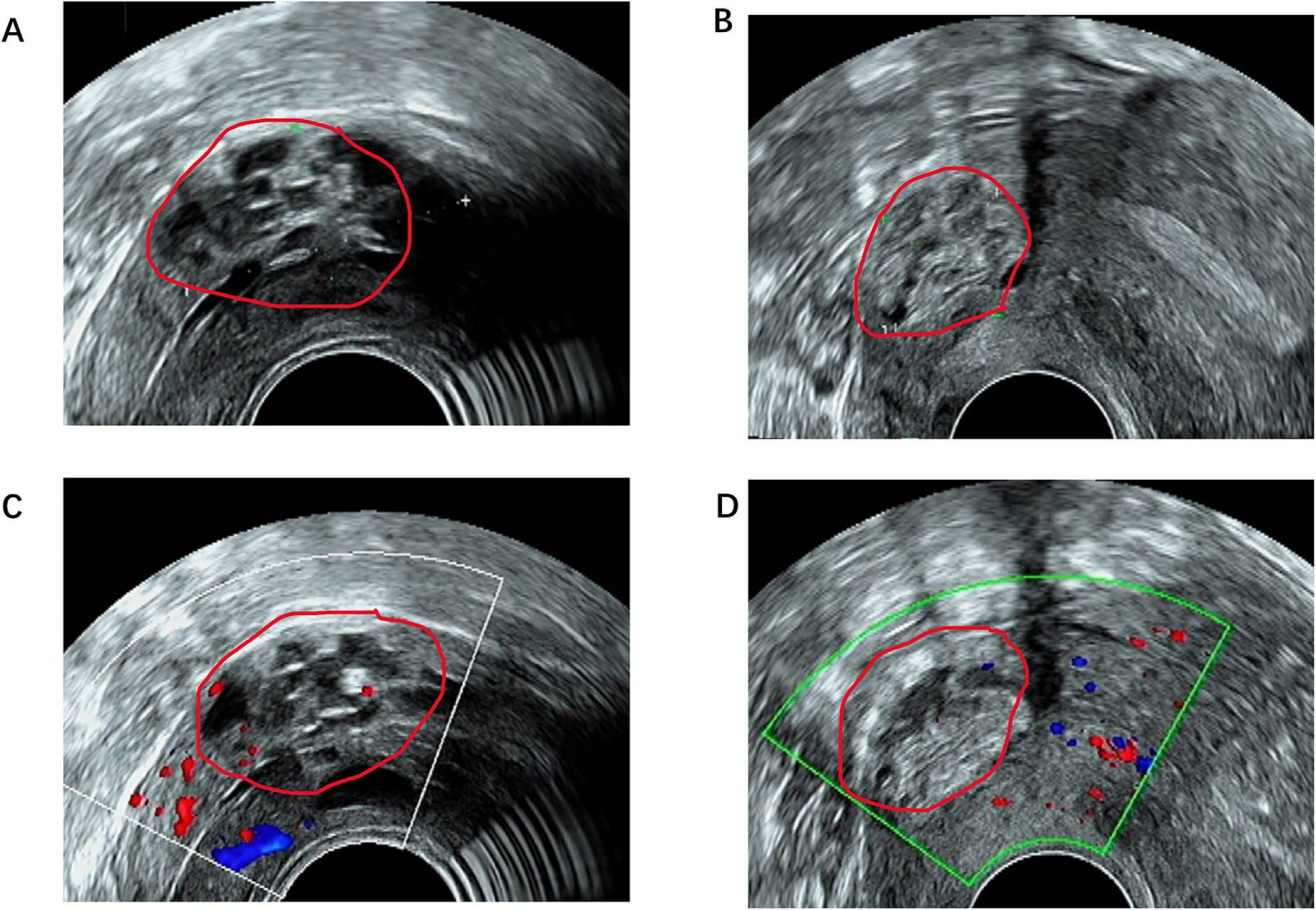
Typical transvaginal sonographic images of LEGH from different planes. A and B showed an area with uneven echoes of various size in the upper part of the cervix. The cystic lesions presented enhanced internal echogenicity and peripheral echo. And the boundary with the surrounding was not clear. C and D revealed absent or sparse blood flow signals.

We conducted a retrospective review of sonographic features of LEGH and documented the characteristic imaging findings. As showed in Figure 2, the sonographic characteristics of the LEGH are summarized in follows. 1) Morphology: Sonographic evaluation demonstrated the location of the lesions predominantly in the upper segment of the cervix. The heterogeneous echogenicity area contained multiple cystic lesions of variable sizes. 2) Echogenicity: LEGH usually presents with enhanced internal echogenicity on ultrasound. 3) Marginal Features: Sonographic evaluation demonstrated obscure boundaries and peripheral echogenic enhancement. These features distinguished them from Nabothian cysts. 4) Vascularity: Doppler ultrasound often reveals absent or sparse blood flow signals within the LEGH lesions. This is a key feature differentiating it from other malignancies. However, due to varying levels of awareness regarding this rare entity among the ultrasound physicians involved, not all characteristic imaging features were consistently archived, precluding a reliable calculation of frequency for each finding. We fully acknowledge this limitation and plan to address it in a future prospective study with standardized imaging protocols and documentation.

## Discussion

Cervical cystic lesions, including Nabothian cysts, LEGH, MDA, and Gastric-type mucinous carcinoma, range from benign to precursor to malignant conditions. These conditions usually present overlapping clinical, pathologic, and imaging manifestations, making it especially challenging to differentiate between them before operation [7]. LEGH has attracted attention as a related precursor lesion for mucinous endocervical adenocarcinomas and MDA based on histopathological features [8]. Small gland proliferation in a lobular fashion, basally located round nuclei without anaplasia, and abundant intracytoplasmic mucin of the glandular epithelium are typical histological characteristics of LEGH [9]. These small glands are often located in the upper portion of the endocervical canal and show the absence of distinct stromal invasion. Atypical LEGH was defined as the presence of LEGH with cytological and architectural atypia without stromal invasion [4]. The risk factors for LEGH have not been clearly elucidated and early detection remains a challenge. LEGH and malignancy associated with LEGH are considered to be HPV- independent endocervical lesions [10]. HPV vaccine does not reduce the risk of LEGH-associated adenocarcinomas. LEGH is considered a metaplastic process with a pyloric gland phenotype, and its incidence is reported to be 0.7% [11]. However, the incidence would probably increase with increasing awareness and more extensive sampling.

As cases of PJS syndrome with LEGH were included, the average patient age was younger than that in previous reports [9]. Among these patients, 85 of 135 presented with watery vaginal discharge, which was consistent with previous reports. In this study, the positive cytological examination rate was only 11.9%, which was significantly lower than that reported in previous studies [6,9]. As LEGH and LEGH-associated malignancies typically occur in the upper part of the endocervical canal, it may be difficult to reach by routine cervical sampling. The consistently high cytology-negative rate in LEGH and related lesions can be attributed to their minimal cytologic atypia, limited spontaneous exfoliation, and frequent location high in the endocervical canal, where routine sampling instruments rarely reach. In real-world practice, this diagnostic pitfall often leads to missed detection or significant underdiagnosis. ‘Targeted endocervical sampling’ could be considered when LEGH or even malignancy was suspected [6]. However, for conventional screening of cervical cancer in larger sample sizes, ‘targeted endocervical sampling’ is not carried out easily. However, awareness of this would be helpful for subsequent histopathological specimen acquisition. Previous cases have also indicated that the distribution of mucin in glandular cells, complex glandular structures, and nuclear atypia should be analyzed together in suspected cases of LEGH [4,9,12,13]. Image quantification technology and immunohistochemical markers have been explored for potential differential diagnosis [14,15]. However, the chronological course of the endocervical cytology of LEGH that develops into malignancy remains unclear.

Diagnostic approaches for LEGH and related disease remain areas of active investigation, yet the overall understanding of LEGH is concurrently deepening. A multicystic lesion located in the upper part of the cervix is easily detected on sonography. The distance between the multicystic lesion and the internal or external cervical os can be measured using transvaginal sonography [6]. Few studies have investigated the sonographic images of LEGH and how they change when LEGH develops. However, ultrasound is a more commonly used tool, and the usefulness of its convenience and imaging location for precise histopathological sampling cannot be disregarded. In this study, we summarized the ultrasonographic characteristics of LEGH. Heterogeneous echogenicity, echogenic enhancement, obscure boundaries, and no significant blood flow signals could be used as indicators for preliminary screening. However, cervical Nabothian cysts usually exhibit anechoic or hypoechoic structures within the cervical stroma, surrounded by normal tissue (no enhanced peripheral echo). The solid components inside and vascularity are particularly important in distinguishing malignancy during patients’ follow-up visits. These findings, derived from our clinical observations, require validation through multi-institutional studies with larger sample sizes. Nevertheless, establishing standardized recognition criteria for the sonographic features of LEGH could significantly enhance the screening efficacy. To describe the shared spectrum of clinical and ultrasound manifestations to enhance the efficiency of initial screening, we conducted a joint analysis of LEGH, atypical LEGH, and malignant cases. Indeed, lumping all the cases together into a single group for the analysis of clinical and imaging features may introduce bias. The absence of a control group with similar imaging findings but different pathologies is a key limitation of this retrospective study, preventing a true assessment of specificity and diagnostic performance.

Owing to its superior soft-tissue contrast and multiplanar capability, MRI is particularly useful for evaluating the complex female pelvis. For diagnosing cystic lesions such as LEGH, MRI provides high diagnostic objectivity and is considered the definitive imaging modality. MRI examination is useful for small lesions and risk stratification of LEGH and malignancy [6,16,17]. According to previous reports [18], MRI findings were classified as follows: solid, invasion, cosmos, microcystic, and coarse cystic patterns. According to a retrospective study, the sensitivity was 73.9% and specificity 84.0% when a combination of ‘cosmos’ or ‘microcystic’ pattern and lesion not circumscribing the cervical canal was present [19]. MRI plays a critical role in the evaluation of cervical lesions by both distinguishing LEGH from other benign cystic entities and identifying related like atypical LEGH prior to their progression to GAS [20]. The combination of MRI, cytological findings, and/or gastric-type mucin assay could certainly improve the predictive accuracy compared with each single examination **[18,21**]. When imaging suggests a lesion in the upper canal despite negative cytology, targeted sampling (e.g. hysteroscopic biopsy and/or conization) may be considered. A predictive model based on age, symptoms, signs, cytology, and ultrasound and MRI characteristics may be possible in the future.

Traditionally, cervical biopsy by vaginoscopy has been the initial approach for histopathological assessment of most cervical lesions. However, for cystic lesions, the diagnosis can easily be missed. The sensitivity of biopsy is only 38.9% [9]. In this study, the detection rate of the vaginoscopic biopsy was 48.5%. It was still not ideal. Cervical conization has been considered to be an important diagnostic approach for LEGH[4]. However, cervical conization is sometimes technically difficult because of the higher site of the lesion, making the surgical approach difficult. Hysteroscopic biopsy is another promising method for diagnosing cervical cystic lesions with the key advantage of direct visualization of the lesion through an endoscope, enabling targeted biopsy of the identified area [22]. We used both hysteroscopic biopsy or cervical conization and a combination method for histopathological diagnosis when the ultrasound, MRI findings, or vaginoscopic biopsy indicated the potential for LEGH or malignancy. The choice of histopathological diagnosis method should be based on imaging findings. Because of the unique location of LEGH, cervical conization combined with hysteroscopic biopsy may be a more valid diagnostic method for LEGH. Classic immunohistochemical markers, including HIK1083, MUC6, and TFF2, are well established for recognizing LEGH and related lesions within the gastric‑type spectrum [14]. Otherwise, CLDN18 (43-14 A) emerged as a potential diagnostic and therapeutic marker for GAS [23]. More recently, HNF4α has emerged as a sensitive adjunct marker across both HPV‑associated and HPV‑independent endocervical glandular lesions, which may prove particularly valuable [24]. However, the calculating sensitivity without data from the entire screened cohort, including true negatives, is a limitation that can introduce selection bias. Further clinical studies with large sample sizes are required to investigate diagnostic accuracy.

LEGH has the potential to develop into cervical malignancy. The results of a previous study indicated that the rate of malignant changes in clinical LEGH was 1.4% (1/69) [18]. However, it may have been underestimated because of its occult characteristics. Whether to remove the uterus is controversial in patients with LEGH patients [21]. Undoubtedly, patients who have given birth are recommended to undergo hysterectomy when carcinoma *in situ* or malignancy is present in pathological results. Traditionally, cervical conization is recommended when the diagnosis is unclear through vaginoscopic biopsy. Notably, whether cervical conization can resect the entire lesion is important for a reserved uterus. There were 12% (12/100) cases underwent pathological upgrading after hysterectomy in this study. According to the imaging findings and location of the lesion, combined with hysteroscopy and cervical conization, it is helpful to completely resect the lesion and preserve the uterus. Laparoscopic surgery has been demonstrated to be feasible for cervical cystic lesions [25]. Techniques to prevent tumor spillage should be considered when performing laparoscopic surgeries. Furthermore, LEGH can occur in patients with PJS [26] and is associated with various tumors that affect the female reproductive system, including cervical GAS and ovarian mucinous neoplasms. PJS patients are usually diagnosed with GAS at an early age. Cervical cystic lesion screening for patients with PJS is easier than that for carcinomas of other organs.

## Conclusion

The detection rate of LEGH has increased significantly in recent years. Early identification of precancerous lesions like atypical LEGH is critical to prevent progression to Gastric-type Adenocarcinoma (GAS), though it remains a clinical challenge. Current practice relies on an integrated assessment utilizing clinical symptoms, cytology, and imaging with ultrasonography for initial screening and contrast-enhanced MRI for superior soft-tissue characterization and differential diagnosis. Heterogeneous echogenicity, echogenic enhancement, obscure boundaries, and absent or sparse blood flow signals are important ultrasonographic indicators for differentiating LEGH. Definitive diagnosis is based on pathological confirmation, and localization of the lesion assists in selecting appropriate pathological diagnostic methods. Of course, multicenter clinical studies with large sample sizes are necessary for guideline development in the future.

## Data Availability

The data that support the findings of this study are available from the corresponding author upon reasonable request. The data are not publicly available due to privacy or ethical restrictions.
